# Improving Procedural Pain Management for Newborns in a Level 3 Neonatal Intensive Care Unit: A Quality Improvement Initiative

**DOI:** 10.7759/cureus.74410

**Published:** 2024-11-25

**Authors:** Sheetal Sriraman, Pavani Chitamanni, Raj Krishna Yadav, Sukhvinder Ranu

**Affiliations:** 1 Neonatology, NewYork-Presbyterian/Columbia University Irving Medical Center, New York, USA; 2 Neonatology, State University of New York Downstate Health Sciences University/Kings County Hospital, New York, USA

**Keywords:** neonates, oral sucrose, pain, pdsa model, quality improvement

## Abstract

Introduction: Neonatal pain has been associated with numerous adverse outcomes, making pain management essential in the neonatal intensive care unit (NICU). Our specific, measurable, actionable, realistic, and timely (SMART) aim was to increase the proportion of neonates receiving pain management interventions during painful procedures from a baseline of less than 30% to above 50% within six months.

Methods: The Plan-Do-Study-Act (PDSA) model for improvement methodology was employed to improve pain management in the NICU between August 2022 and July 2023. Interventions included educational campaigns, monthly meetings, weekly huddles, smart order sets, readily available medication information, and accessibility. Data was collected by reviewing the electronic health record (EHR), and survey data was collected through anonymous surveys of the NICU staff. The outcome measures were the proportion of sucrose and 4% lidocaine orders placed and the proportion of procedures that received pain management interventions.

Results: During the period from August 2022 to July 2023, there were 383 newborns admitted to the NICU. On average, each newborn underwent 42.8 procedures during their hospital stay. The proportion of neonates who were ordered sucrose and 4% lidocaine increased from a baseline of 7% and 21% to 43% and 54% after the second PDSA cycle, respectively. The proportion of procedures for which sucrose and lidocaine were administered increased from 16% and 21% at baseline to 54% and 65% after the second PDSA cycle.

Conclusion: The quality improvement (QI) methodology in conjunction with the education of NICU staff and the incorporation of smart order sets can be used to effectively increase the use of pain management interventions in the NICU.

## Introduction

Neonatal pain management is a critical aspect of neonatology. Newborns undergo more than 300 painful procedures and surgeries during their hospital stays [1,2]. Evidence suggests that not only are neonates capable of experiencing pain but most of them are hypersensitive to pain due to their immature nervous systems [3,4].

While the current consensus dictates that healthcare providers should incorporate pain management measures for neonates undergoing painful procedures, in reality, the management of procedural pain management in neonates differs significantly [5,6]. Continuous pain monitoring is conducted for only around 10% of neonates admitted to the neonatal intensive care unit (NICU) [7]. Cruz et al. demonstrated that neonates undergo 7.5-17.3 painful procedures daily and often receive inadequate pain management during these procedures [2]. Simons et al. found that infants undergo an average of 14 painful procedures daily, with fewer than 35% receiving procedural pain management on each study day [8].

Studies indicate that neonatal pain is linked to several long-term adverse outcomes [9,10]. Neonatal pain has been associated with a reduced cortisol response to stress, lower body weight at 32 corrected weeks, and changes in cortical thickness in children at seven years of age [11,12]. Furthermore, neonatal pain is linked to a decreased pain threshold in both animal models and premature infants [13]. Hermann et al. demonstrated that pain in the neonatal period leads to enhanced sensitization to painful stimuli during school age [14]. Neonatal pain is also correlated with poorer cognitive and motor development later in life [15]. Hunt et al. showed that infants who underwent surgical procedures had an increased incidence of major neurosensory disability [16]. Additionally, neonatal pain and injury are associated with reduced cerebellar, amygdala, and thalamus volumes, resulting in poorer cognition and behavioral outcomes for children [17,18]. Steinbauer et al. showed that an increased painful stimulus during the neonatal period was associated with neuronal cell loss in rodent models [19].

At our institution, there was a lack of understanding of neonatal pain and an absence of routine administration of neonatal pain management interventions. Recognizing the significance of neonatal pain management, we initiated a quality improvement (QI) project to improve procedural pain management measures in our NICU. Reviewing baseline survey responses revealed that the main barriers to implementing pain management interventions in the NICU among healthcare staff were a lack of understanding of pain management, increased time to administer pain management, and lack of accessibility to pain management interventions. Therefore, interventions focused on education of NICU staff, making sucrose and lidocaine more accessible to NICU staff and incorporating smart order sets which made it easier for providers to order pain management intervention for patients.

Our specific, measurable, actionable, realistic, and timely (SMART) aim was to increase the proportion of neonates receiving pain management interventions during painful procedures from a baseline of less than 21% to above 50% within six months.

## Materials and methods

Setting

This QI initiative was conducted at New York City Health+Hospitals/Kings County's NICU. The hospital serves as both a community hospital and a referral center for infants within the New York City Health+Hospitals System in Brooklyn. The NICU is classified as a level 3 unit and accommodates 30 beds. The unit typically maintains an average census of 15-20 infants. The hospital's primary workforce consists of attending neonatologists, neonatal fellows, resident physicians, registered nurses, neonatal nurse practitioners, neonatal hospitalists, and patient care associates. Additionally, the unit operates as a teaching facility, with medical and physician assistant students regularly completing rotations within the unit. Most of the bedside procedures within the unit are performed by registered nurses and resident physicians, under the supervision of attending neonatologists, collectively referred to as NICU staff in this study.

Study design

**Figure 1:**
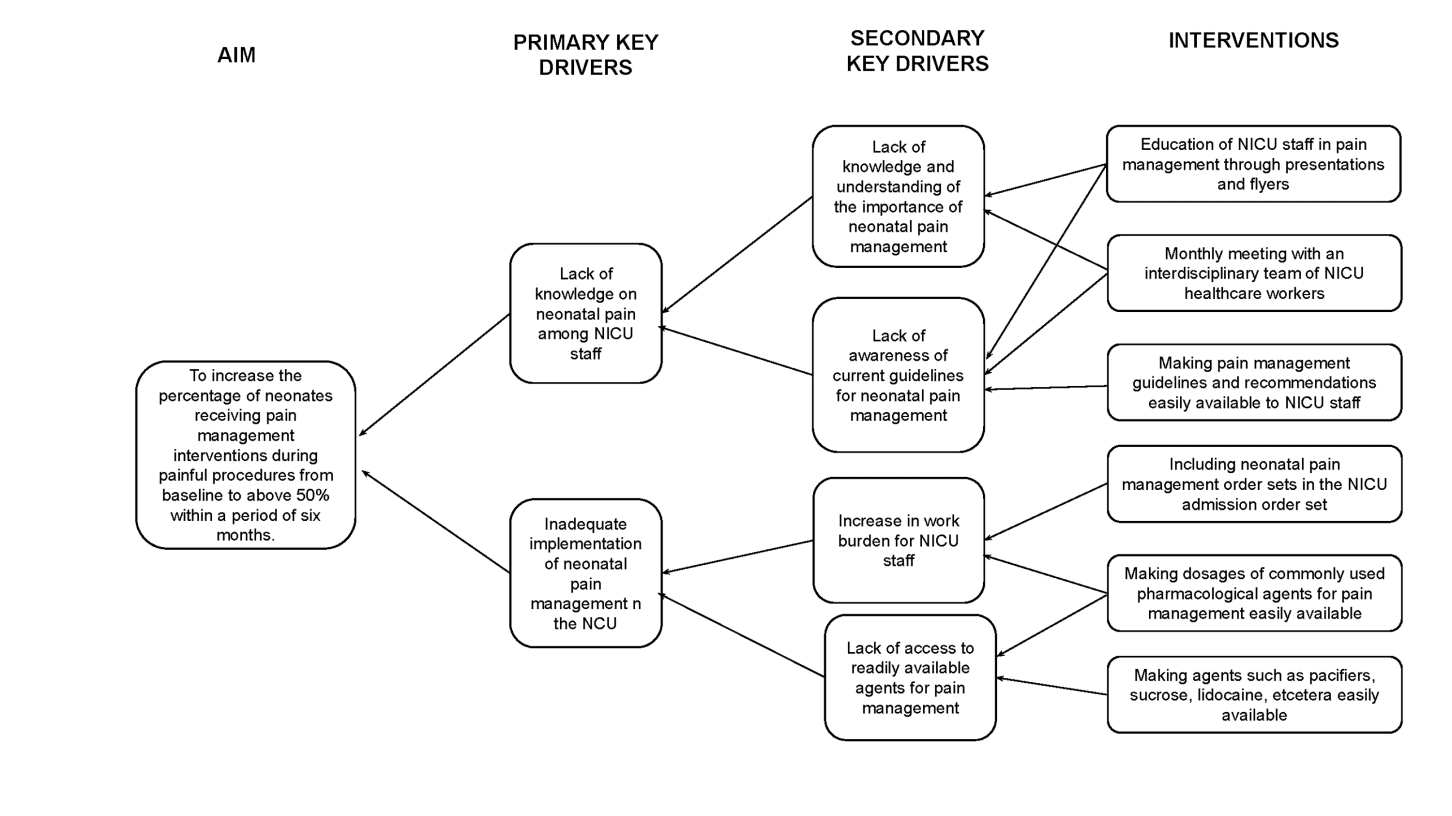
Key driver diagram used during the planning and implementation phases of the QI QI: quality improvement

**Figure 2:**
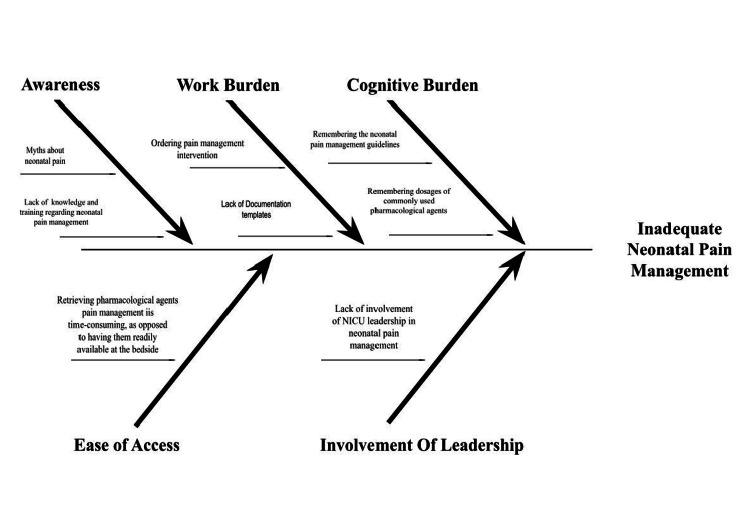
Fishbone diagram

The Plan-Do-Study-Act (PDSA) model for improvement methodology was employed to address the low rates of neonatal pain management in the NICU. The QI team was composed of resident and attending physicians. An interdisciplinary team of stakeholders and advisers included attending physicians, resident physicians, registered nurses, patient care associates, and pharmacists. To guide the planning and implementation of the QI initiative, a key driver and fishbone diagrams were used (Figure 1 and Figure 2). A detailed description of various interventions during the first and second PDSA cycles is presented in Table 1.

**Table 1:** Description of interventions during the first and second PDSA cycles PDSA: Plan-Do-Study-Act; NICU: neonatal intensive care unit; QI: quality improvement

Intervention	Description
First PDSA cycle	
Educational campaign	An educational campaign was initiated to educate residents and nurses about neonatal pain management, along with current evidence-based recommendations and practices. These educational sessions were conducted in person through the use of PowerPoint presentations. Subsequently, the PowerPoint presentations were distributed to the NICU staff via email, and physical copies were provided to those who preferred them. Educational flyers on pain management were prominently displayed in high-traffic areas within the NICU, including the resident workroom, nurse's lounge, and nurse's workstation.
Monthly stakeholder meetings	Monthly meetings were held with a multidisciplinary team, consisting of attendings, residents, nurses, patient care associates, and pharmacists, to deliberate on the QI goals and identify barriers to implementing pain management interventions.
Weekly huddles	Weekly huddles were conducted with the QI team and NICU staff to reinforce the QI goals, discuss obstacles, and brainstorm strategies to overcome them.
Note-writing assistance	To assist NICU staff in documenting the pain management modality, smart phrases for common procedures were integrated into the electronic medical record template.
Readily available medication information	Information regarding dosages, routes of administration, and indications for frequently used pain management medications, such as sucrose, subcutaneous lidocaine, acetaminophen, morphine, and fentanyl, was made easily accessible in resident workrooms.
Second PDSA cycle	
Smart order sets	Smart order sets were developed and incorporated into the electronic medical record system. These included the comfort bundle as part of the routine neonatal order set. The comfort bundle comprised orders for comfort positions, numbing, distraction, breastfeeding when applicable, lidocaine cream, 24% sucrose oral solution, swaddling, and gentle handling.
Improved accessibility	Commonly used pain management tools, including pacifiers, sucrose solution, lidocaine cream, and lubricant jelly, were placed in close proximity to the infant's incubators or bassinets to enhance accessibility.

Painful procedures in the context of this study encompass capillary or heel sticks, venous puncture, arterial puncture, intravenous line placement, peripherally inserted central catheter (PICC) line placement, adhesive removal, bladder catheterization, and nasogastric or orogastric tube insertion. Non-pharmacological pain management interventions in this study comprise positions of comfort, swaddling, gentle handling, numbing, distraction, oral sucrose solution, and kangaroo care or breastfeeding when appropriate. Pharmacological pain management interventions include the use of 4% lidocaine cream.

The study obtained an exempt status from the Institutional Review Board (IRB) at the State University of New York (SUNY) Downstate Medical Center (IRB ID: 1952090-1).

Phases

The study proceeded in the following phases:

Pre-intervention Phase

Baseline data was collected between the months of August and September 2022.

Intervention Phase

The first PDSA cycle was conducted between October 2022 and December 2022, while the second PDSA cycle was conducted between January 2023 and March 2023.

Post-intervention Phase

The maintenance phase of the study was between April 2023 and July 2023.

Interventions

Interventions to implement were decided based on the responses to the baseline survey of NICU staff. Lack of awareness about neonatal pain management and lack of education regarding neonatal pain management interventions were identified as one of the main barriers. Therefore, the interventions in the first PDSA cycle focused on education. The following interventions were implemented in the first PDSA cycle.

Educational Campaign

An educational campaign was initiated to educate residents and nurses about neonatal pain management, along with current evidence-based recommendations and practices. These educational sessions were conducted in person through the use of PowerPoint presentations. Subsequently, the PowerPoint presentations were distributed to the NICU staff via email, and physical copies were provided to those who preferred them. Educational flyers on pain management were prominently displayed in high-traffic areas within the NICU, including the resident workroom, nurse's lounge, and nurse's workstation.

Monthly Stakeholder Meetings

Monthly meetings were held with a multidisciplinary team, consisting of attendings, residents, nurses, patient care associates, and pharmacists, to deliberate on the QI goals and identify barriers to implementing pain management interventions.

Weekly Huddles

Weekly huddles were conducted with the QI team and NICU staff to reinforce the QI goals, discuss obstacles, and brainstorm strategies to overcome them.

Note-Writing Assistance

To assist NICU staff in documenting the pain management modality, smart phrases for common procedures were integrated into the electronic medical record template.

Readily Available Medication Information

Information regarding dosages, routes of administration, and indications for frequently used pain management medications, such as sucrose, subcutaneous lidocaine, acetaminophen, morphine, and fentanyl, was made easily accessible in resident workrooms.

For the second PDSA cycle, feedback from the first PDSA cycle was incorporated. Based on the feedback from the NICU staff, smart order sets were developed and incorporated into the electronic medical record system. These included the comfort bundle as part of the routine neonatal order set. The comfort bundle comprised orders for comfort positions, numbing, distraction, breastfeeding when applicable, lidocaine cream, 24% sucrose oral solution, swaddling, and gentle handling. Commonly used pain management tools, including pacifiers, sucrose solution, lidocaine cream, and lubricant jelly, were placed in close proximity to the infant's incubators or bassinets to enhance accessibility.

Data collection process

Order set data for sucrose and lidocaine was collected through reports created in the electronic medical record. To determine the number of procedures performed and if pain management was administered, a review of individual infant charts was performed. Data for the survey of the NICU staff was collected via an anonymous survey. The surveys were then retrieved by the QI team at three specified times during the week. Throughout the data collection process, discretion and anonymity were meticulously maintained. Data for the process measure was collected through records maintained by the QI team following each presentation. Data for the balancing measure was obtained through surveys conducted during the first and second PDSA cycles.

Measures

Outcome Measure

The primary outcome measures were the proportion of infants in whom sucrose and topical lidocaine were placed and the proportion of procedures for which pain management interventions were administered. Data was collected through reports created in the electronic medical record to determine the number of orders placed and review of individual infant charts to determine the number of procedures performed.

The secondary outcome measure included the percentage of times that NICU staff reported implementing pain management in the NICU. Data for this measure were gathered through surveys administered to the NICU staff.

Process Measure

The process measure entailed quantifying the number of NICU staff members who underwent training and education on neonatal pain management, along with current evidence-based recommendations and practices.

Balancing Measure

Balancing measures encompassed the potential increase in workload for NICU staff resulting from the administration of pain management interventions and any potential delays in neonatal care due to such interventions.

Statistical analysis

The proportion of infants for whom sucrose and lidocaine orders were placed and the proportion of procedures for which pain management interventions were administered are represented in the form of a statistical process control (SPC) chart. The percentage of NICU staff reporting the use of pain management interventions during painful procedures more than 70% of the time was determined based on survey responses. To analyze the pre- and post-intervention survey responses, the chi-squared test for categorical variables was employed. A p-value of <0.05 was deemed statistically significant. Statistical analysis was carried out using Python (Python Software Foundation, Fredericksburg, Virginia, United States).

## Results

**Figure 3:**
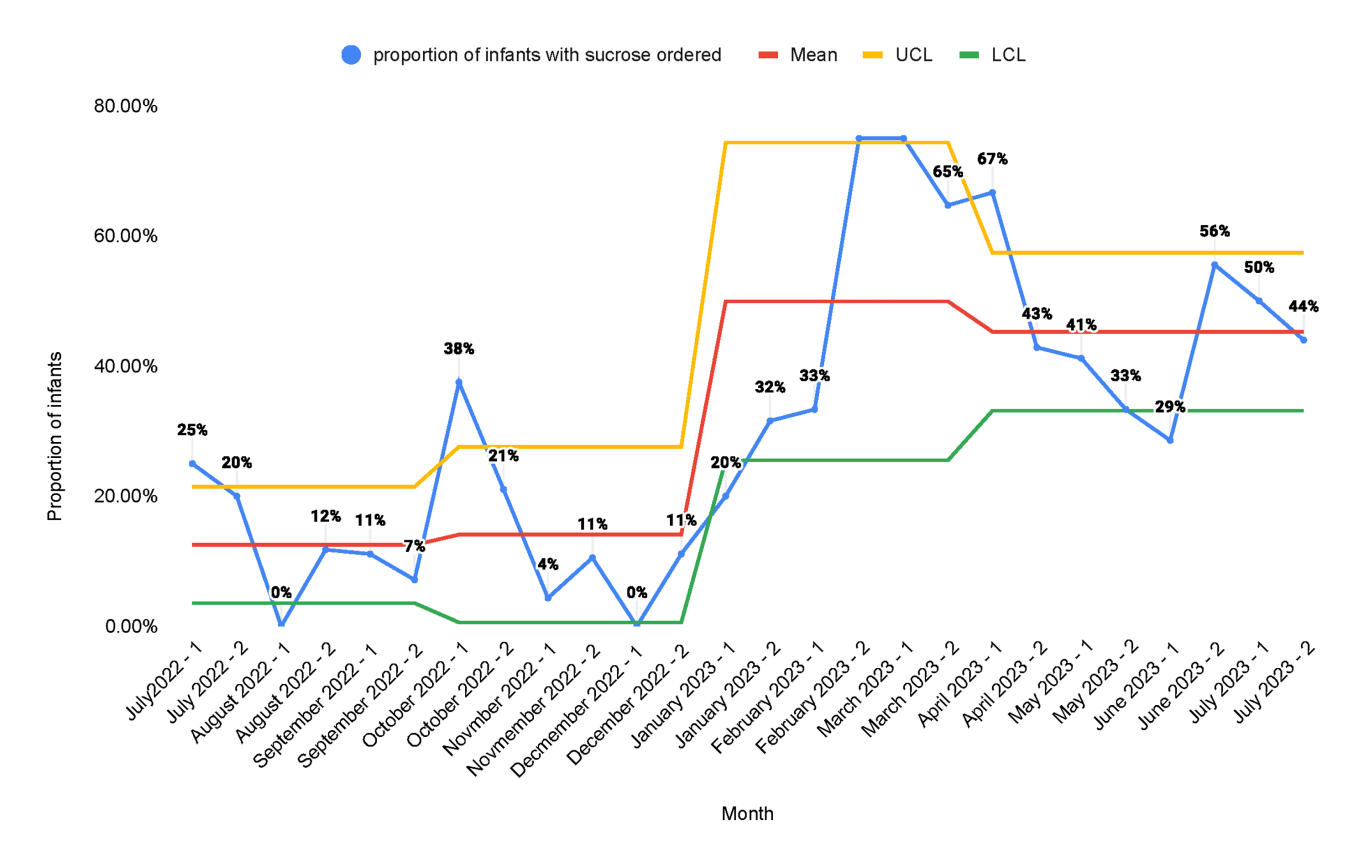
Statistical process control chart representing the proportion of infants in the NICU who were ordered oral sucrose solution A: educational campaign; B: monthly stakeholder meetings; C: weekly huddles; D: note-writing assistance; E: readily available medication information; F: incorporation of smart order sets; G: placement of sucrose and lidocaine in readily accessible locations NICU: neonatal intensive care unit; UCL; upper control limit; LCL: lower control limit

During the period from July 2022 to July 2023, there were 382 newborns admitted to the NICU. On average, each newborn underwent 42.4 procedures for the duration of their hospital stay. The proportion of neonates in whom sucrose orders were placed increased from a baseline of 12.5% to 45.3% after the second PDSA cycle (Figure 3).

**Figure 4:**
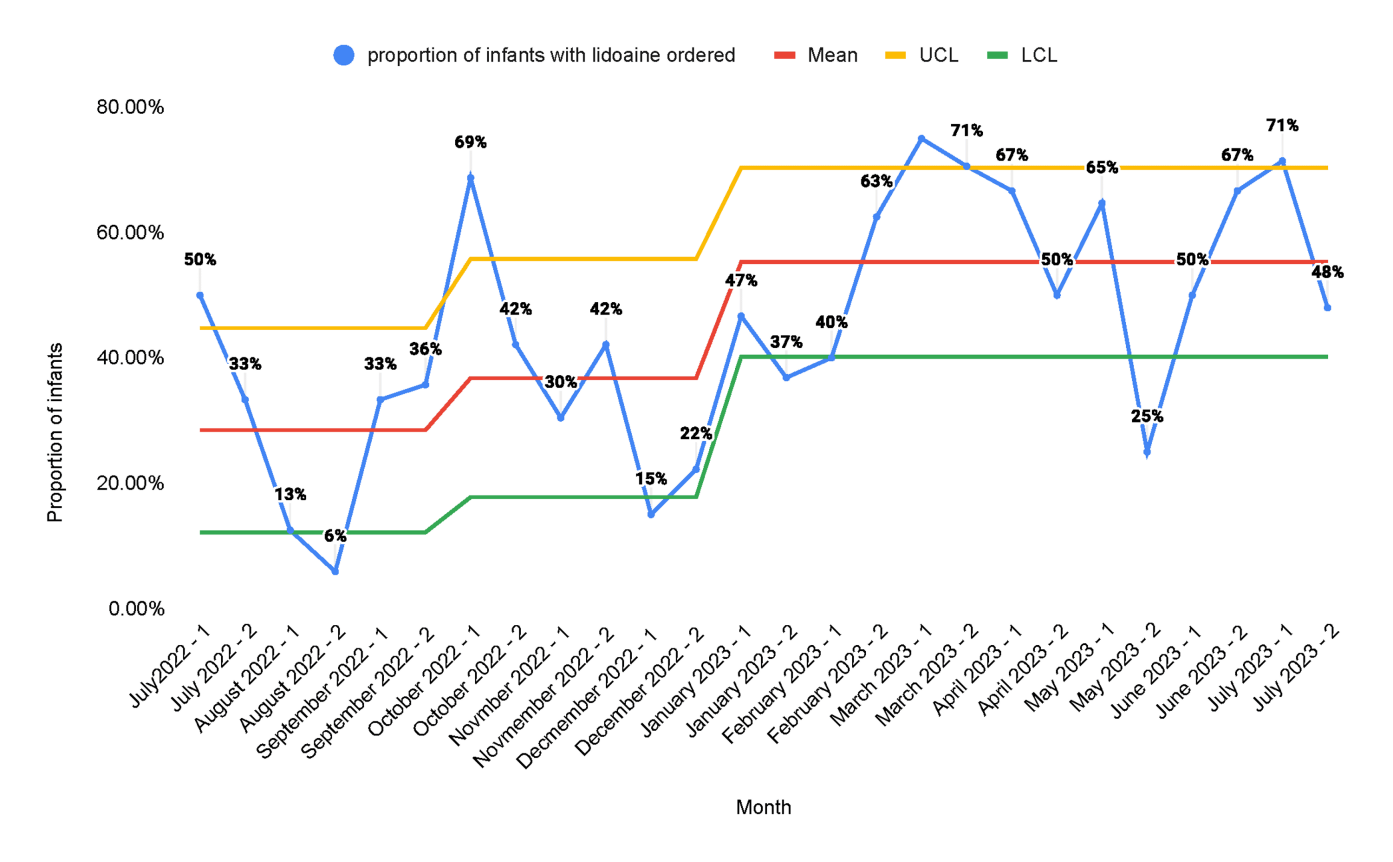
Statistical process control chart representing the proportion of infants in the NICU who were ordered 4% lidocaine A: educational campaign; B: monthly stakeholder meetings; C: weekly huddles; D: note-writing assistance; E: readily available medication information; F: incorporation of smart order sets; G: placement of sucrose and lidocaine in readily accessible locations NICU: neonatal intensive care unit; UCL: upper control limit; LCL: lower control limit

The proportion of neonates in whom lidocaine orders were placed increased from a baseline of 28.4% to 55.3% after the second PDSA cycle (Figure 4).

**Figure 5:**
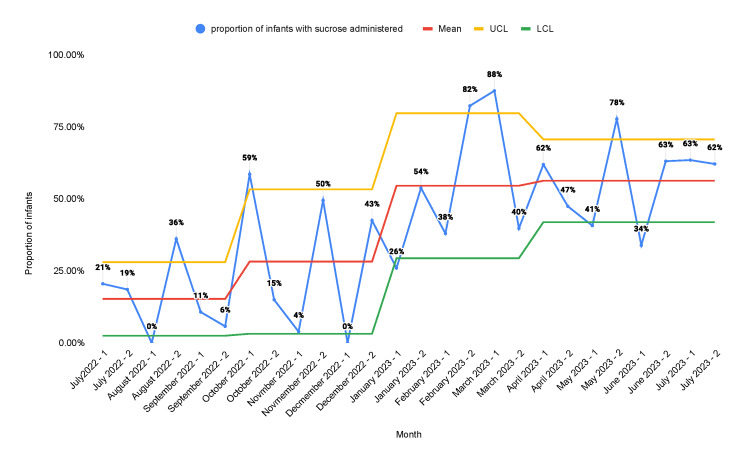
Statistical process control chart representing the proportion of procedures that received sucrose for pain A: educational campaign; B: monthly stakeholder meetings; C: weekly huddles; D: note-writing assistance; E: readily available medication information; F: incorporation of smart order sets; G: placement of sucrose and lidocaine in readily accessible locations UCL: upper control limit; LCL: lower control limit

The proportion of procedures performed for which infants received sucrose was 15.2% at baseline and increased to 56.3% after the second PDSA cycle (Figure 5).

**Figure 6:**
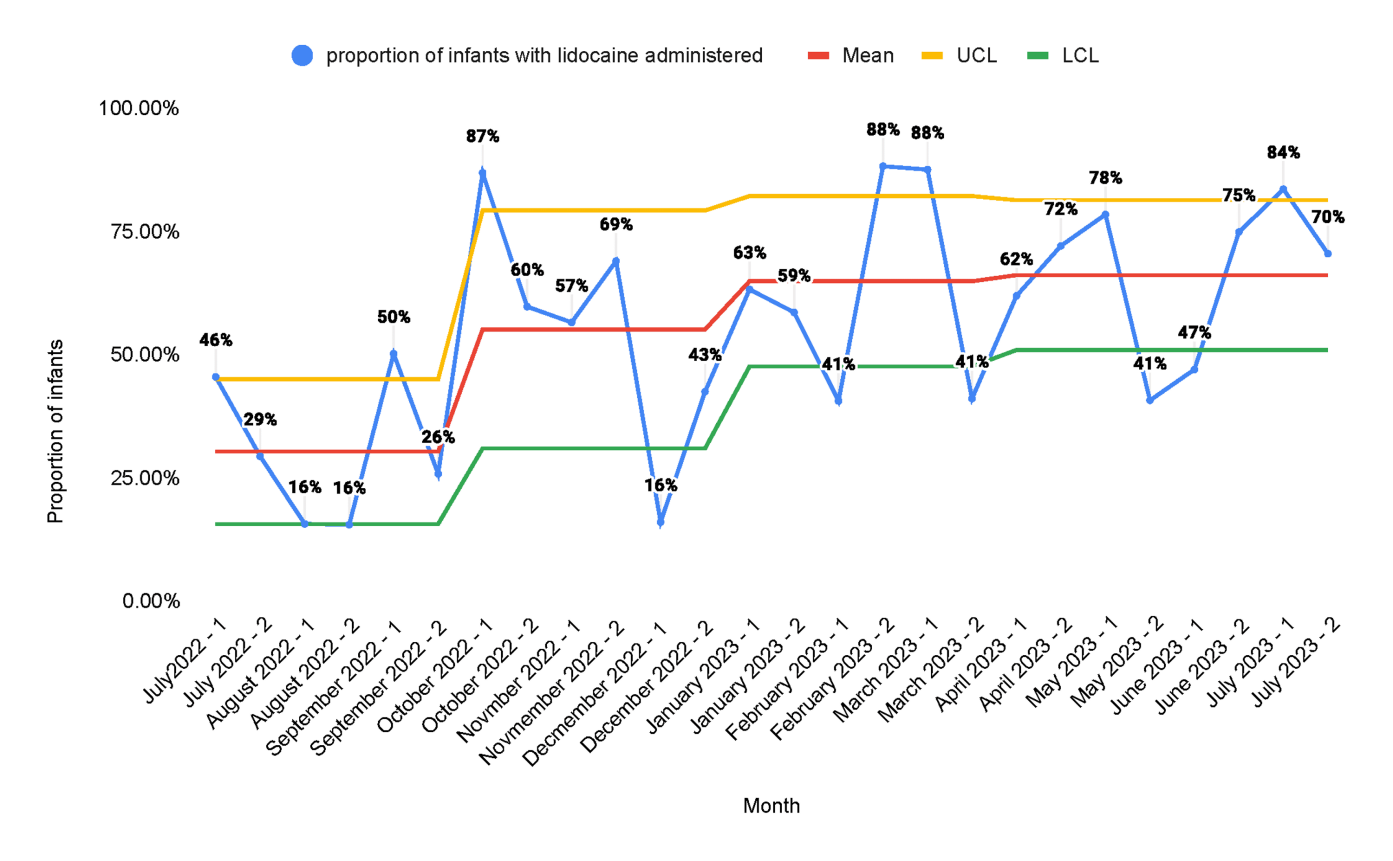
Statistical process control chart representing the proportion of procedures that received lidocaine for pain management A: educational campaign; B: monthly stakeholder meetings; C: weekly huddles; D: note-writing assistance; E: readily available medication information; F: incorporation of smart order sets; G: placement of sucrose and lidocaine in readily accessible locations UCL: upper control limit; LCL: lower control limit

The proportion of procedures performed for which infants received lidocaine was 30.3% at baseline and increased to 66.1% after the second PDSA cycle (Figure 6).

A total of 61 healthcare staff were included in the baseline pre-intervention survey, comprising 40.9% (25/61) residents and 59% (36/61) nurses. Approximately 40% of the residents were first-year residents, and 60% of the residents were second-year residents. Over half of all nurses had between one and 10 years of experience as neonatal nurses. Over 75% of all healthcare staff in the NICU reported performing between one and five procedures per day.

The number of healthcare staff who believed that neonates were capable of feeling pain was 95.1% (58/61) at baseline and 100% during the first and second PDSA cycles, respectively. The number of healthcare staff who believed that pain management is necessary in the NICU was 86.9% (53/61) at baseline and 100% during the first and second PDSA cycles, respectively.

The percentage of NICU staff who reported implementing pain management for various painful procedures in the NICU significantly improved from baseline at the end of the second PDSA cycle, as evident from Table 2.

**Table 2:** Results of the staff survey reporting using pain management interventions during the pre-intervention phase, first and second PDSA cycles The table reports the number (percentage) of NICU staff who have reported following the specified neonatal pain management interventions at least 70% of the time The chi-squared test for equality of proportions was used for statistical analysis. A p-value <0.05 was considered to be statistically significant NICU: neonatal intensive care unit; PDSA: Plan-Do-Study-Act

Interventions	Pre-intervention baseline (n=61)	First PDSA (n=36)	Second PDSA (n=39)	Chi-squared value	P-value
Use of non-pharmacological measures during skin-breaking procedures ≥70% of the time	36 (59%)	30 (83.8%)	36 (92.3%)	15.88	<0.001
Use of topical anesthetic agents during skin-breaking procedures ≥70% of the time	3 (4.9%)	19 (52.8%)	22 (56.4%)	38.15	<0.001
Use of topical anesthetic agents or lubricants during oro-/nasogastric tube placement or catheterization ≥70% of the time	6 (9.8%)	17 (47.2%)	27 (69.2%)	38.40	<0.001
Use of measures to decrease pain during adhesive removal	19 (31.1%)	31 (86.1%)	37 (94.9%)	52.33	<0.001
NICU staff who reported to be adequately trained in pain management	9 (14.8%)	22 (61.1%)	33 (84.6%)	50.49	<0.001

Over half of the NICU staff reported requiring less than one minute to administer non-pharmacological pain management interventions. Most of the NICU staff reported requiring 1-5 minutes to administer pharmacological pain management interventions. The number of NICU staff who reported that instituting the pain management interventions did not increase their workload was 52.8% (19/36) and 82% (32/39) during the first and second PDSA cycles. Most NICU staff reported that instituting pain management interventions did not delay patient care in any way in PDSA cycles. The most commonly reported barriers to instituting neonatal pain management interventions were the availability of pharmacological agents, the time taken to implement the intervention, and the time taken to bring the pharmacological agent to the bedside.

## Discussion

In the present study, most healthcare staff believed that neonates experience pain and that pain management is necessary in the NICU. These findings are supported by existing literature. Agakidou et al. conducted a two-point survey, showing that all healthcare workers surveyed in 2000 and 2019 believed that neonates are capable of feeling pain [20]. Carlsen Misic et al. found that in a survey of over 200 nurses, 91% of nurses reported that pain management was important, but only 53% reported following current pain management guidelines [21]. Ishak et al. conducted a survey among physicians and found that those with more experience had a better knowledge of neonatal pain and management practices [22].

Numerous studies have explored optimal neonatal pain management strategies. Current recommendations for pain management involve a stepwise escalation of interventions based on the type of painful procedure and neonatal pain scores. Both non-pharmacological and pharmacological interventions for routine bedside procedures in term and preterm neonates are encouraged. Non-pharmacological strategies encompass oral sucrose, non-nutritive sucking via pacifier, kangaroo care, skin-to-skin contact, and swaddling, among others. Pharmacological interventions include the use of topical analgesics like lidocaine, acetaminophen, and opioids [5,6]. Notably, although manufacturer recommendations may not include preterm neonates as the target population for topical analgesics, several studies have demonstrated their safe and efficacious use during skin-breaking procedures in both term and preterm neonates [23,24]. Kaur et al. demonstrated that 4% lidocaine topical anesthetic agents reduced pain during venipunctures in both term and preterm neonates [23]. A randomized controlled trial by Kaur et al. showed the effectiveness of topical EMLA in reducing pain scores during lumbar punctures [24].

Multiple QI studies have been done to enhance neonatal pain management. Anne et al. published a quality report in 2021 that demonstrated an increase in the administration of pain management interventions from a baseline of 13% to 73% [25]. Our study produced similar results. The proportion of neonates in whom sucrose orders were placed increased from a baseline of 12.5% to 45.3%. Similarly, the proportion of neonates in whom lidocaine orders were placed increased from a baseline of 28.4% to 55.3%. The proportion of procedures performed for which infants received sucrose was 15.2% at baseline and increased to 56.3% after the second PDSA cycle. The proportion of procedures performed for which infants received lidocaine was 30.3% at baseline and increased to 66.1% after the second PDSA cycle. There was a decrease in the number of orders placed and the number of procedures that received pain management interventions in the second half of the first PDSA cycle. We believe that this was due to the fact that the main intervention in the first PDSA cycle was education. After the introduction of other measures such as sucrose and lidocaine orders in the admission order set and making the medications more readily accessible at the bedside, we found sustained improvement. This shows that the education of healthcare staff alone is not reliable as a sustainable intervention for improvement.

There was a statistically significant increase in reported NICU staff using non-pharmacological and pharmacological pain management interventions in the first PDSA cycle with further increases in the second PDSA cycle. Sawleshwarkar et al. achieved similar results in their study, where the rate of sucrose administration increased from 0% to 96% [26]. Many other studies have aimed to improve procedural pain management in the NICU with varying degrees of success [27,28]. Improvement in the use of topical lubricant gel during orogastric and nasogastric tube insertions was unique to our study. Additionally, there was an increase in the use of pain management interventions during adhesive removal, a metric not currently described in existing literature.

In the study, the most common barriers to neonatal pain management interventions were the availability of pharmacological agents, the time required for implementation, and the time taken to bring the pharmacological agent to the bedside. Byrd et al., in a study among nurses, found that the primary barriers to neonatal pain management were inadequate staff training [29]. In 2023, Neshat et al. described various organizational challenges in neonatal pain management, including poor collaboration, lack of protocols, and increased workload for healthcare staff [30].

Our study revealed that after the second PDSA cycle, the proportion of infants who had sucrose orders placed was 45.3% and the proportion of procedures for which sucrose was administered was 56.3%. However, according to self-reported assessments, over 90% of NICU staff reported utilizing pain management interventions in more than 70% of the procedures they perform. This variance could be attributed to the inclusion of various non-pharmacological pain management modalities, such as positioning for comfort, swaddling, gentle handling, numbing, distraction, and kangaroo care or breastfeeding, in addition to oral sucrose administration. Similarly, the proportion of infants in whom lidocaine orders were placed was 55.3%, and the proportion of procedures for which lidocaine was administered was 66.1%. This finding aligns with the response from the survey where 56.4% of NICU staff reported administering topical lidocaine for more than 70% of skin-breaking procedures.

Our study focuses on an interdisciplinary, collaborative team approach to improve the knowledge and implementation of procedural pain management and contributes to existing literature. The limitations of our study include the fact that the study was a single-center study in a level 3 NICU with 30 beds, making it not readily generalizable. The study is limited to bedside procedures only and did not explore pain management interventions during other painful procedures like intubations or chest tube placements, as these are less frequently performed in the unit. In our unit, sucrose and lidocaine are now available at the bedside, and nurses are not required to document the completion of sucrose or lidocaine orders after each procedure, as these are standing orders and are part of the admission order set. Therefore, determining the accuracy of order completion was challenging. However, we confirmed the usage of sucrose single-use vials and lidocaine from the pharmacy log. Additionally, the secondary outcome of the study was the self-reported survey by the NICU healthcare staff. Given the self-reported nature of the survey, it should be interpreted with caution. This potential bias was partially mitigated by ensuring that both pre- and post-intervention surveys were completely anonymous.

## Conclusions

The use of QI methodology resulted in the increased utilization of procedural pain management interventions in all areas examined. The interdisciplinary team approach, coupled with the QI methodology, proves effective in enhancing the knowledge and the application of procedural pain management within the NICU. In our QI, incorporating smart order sets and making sucrose and lidocaine more readily accessible aided in the implementation of pain management interventions and the sustainability of the QI. The primary barriers frequently cited for the implementation of pain management interventions encompassed the availability of pain management agents, the time required for intervention administration, and the time taken to deliver the agent to the bedside. The authors intend to expand upon this study by assessing pain scores before and after the application of procedural pain management within the unit.
